# CoVaCS: a consensus variant calling system

**DOI:** 10.1186/s12864-018-4508-1

**Published:** 2018-02-05

**Authors:** Matteo Chiara, Silvia Gioiosa, Giovanni Chillemi, Mattia D’Antonio, Tiziano Flati, Ernesto Picardi, Federico Zambelli, David Stephen Horner, Graziano Pesole, Tiziana Castrignanò

**Affiliations:** 1 Dipartimento di Bioscienze, Università degli Studi di Milano, Milan, Italy; 2 SCAI, Cineca, Consorzio Interuniversitario di Supercalcolo, Rome, Italy; 3 Istituto di Biomembrane Bioenergetica e Biotecnologie Molecolari, Consiglio Nazionale delle Ricerche, Bari, Italy; 4 Dipartimento di Bioscienze, Biotecnologie e Biofarmaceutica, Università degli Studi di Bari “A. Moro”, Bari, Italy

**Keywords:** Variant calling, Web server, Workflow, Consensus method, Graphical user interface, Variant annotation, Variant prioritization

## Abstract

**Background:**

The advent and ongoing development of next generation sequencing technologies (NGS) has led to a rapid increase in the rate of human genome re-sequencing data, paving the way for personalized genomics and precision medicine. The body of genome resequencing data is progressively increasing underlining the need for accurate and time-effective bioinformatics systems for genotyping - a crucial prerequisite for identification of candidate causal mutations in diagnostic screens.

**Results:**

Here we present CoVaCS, a fully automated, highly accurate system with a web based graphical interface for genotyping and variant annotation. Extensive tests on a gold standard benchmark data-set -the NA12878 Illumina platinum genome- confirm that call-sets based on our consensus strategy are completely in line with those attained by similar command line based approaches, and far more accurate than call-sets from any individual tool. Importantly our system exhibits better sensitivity and higher specificity than equivalent commercial software.

**Conclusions:**

CoVaCS offers optimized pipelines integrating state of the art tools for variant calling and annotation for whole genome sequencing (WGS), whole-exome sequencing (WES) and target-gene sequencing (TGS) data. The system is currently hosted at Cineca, and offers the speed of a HPC computing facility, a crucial consideration when large numbers of samples must be analysed. Importantly, all the analyses are performed automatically allowing high reproducibility of the results. As such, we believe that CoVaCS can be a valuable tool for the analysis of human genome resequencing studies. CoVaCS is available at: https://bioinformatics.cineca.it/covacs.

**Electronic supplementary material:**

The online version of this article (10.1186/s12864-018-4508-1) contains supplementary material, which is available to authorized users.

## Background

The increasing throughput and reduction in costs associated with Next Generation Sequencing technologies (NGS) is driving personalized genomics and predictive medicine [1–3]. National Health agencies and institutions worldwide are currently undertaking ambitious sequencing projects, aimed to determine the genotypes of tens or even hundreds of thousands of individuals [4–7], to assist in the development of diagnostic approaches and clinical screening programs. The widespread application of such technologies promises major advances in medical science.

While the ability to sequence an unprecedented number of human genomes could serve as the basis for a new revolution in medical science and genetics, the need to handle, analyze and store huge amounts of data is posing major challenges to genomics and bioinformatics, which at present, remain largely unresolved [8]. A presumably incomplete catalog of NGS sequencing platforms (http://omicsmaps.com/) suggests that 2200 NGS instruments are distributed, worldwide. in 1027 sequencing facilities across 62 countries. A conservative estimate based on these numbers suggests that, if used at full capacity, these NGS platforms could generate in the excess of 35 petabases of sequencing data per year [9], while, worldwide, sequencing capacity could be expected to reach zettabases in the next ten years, corresponding to 100 million to 2 billions complete human genome sequences by 2027.

While bioinformatics approaches for the analysis and storage of contemporary sequence data have evolved in parallel with the sequencing technologies themselves, the construction of dedicated pipelines to call sequence variants from different types of data (gene panels, exomes, or whole genomes) and from distinct sequencing technologies, can be technically challenging [10] and typically requires intervention by dedicated bioinformaticians [11, 12]. Furthermore different combinations of tools can yield widely differing results from the same data, complicating direct comparisons [13] and posing a major challenge for the integration of such data into curated databases of human genetic variation [14, 15]. Recent studies, indicating that consensus call-set approaches from different methods are generally more robust and accurate than genotyping strategies based on individual tools (e.g. [16]), also imply a substantial increase in required computational and technical resources. Moreover, strategies based on the combination of different tools result in a rather complex workflow, which can be difficult to implement and optimize, especially for users with a limited bioinformatics background.

In this paper, we present CoVaCS (Consensus Variant Calling System), a fully automated system for genotyping and variant annotation of resequencing data produced by second generation NGS technologies. CoVaCS offers state of the art tools for variant calling and annotation along with an expert made pipeline for the analysis of whole genome shotgun (WGS), whole exome sequencing (WES) and targeted resequencing data (TGS), performing all steps from quality trimming of the sequencing data to variant annotation and visualization. The final set of variants is obtained by forming a consensus call-set (2 out of 3 rule) from three different algorithms based on complementary approaches: Varscan, [17] which adopts a series of stringent quality metrics in order to identify putative false positive predictions, GATK, [18, 19] which performs local reassembly of the reads to mitigate sequence errors and reconstruct haplotypes and Freebayes [20] which is based on a probabilistic haplotype reconstruction algorithm. Extensive tests on a golden standard benchmark based on the NA12878 Illumina platinum genome, confirm that call-sets based on our consensus strategy are completely in line with those attained by similar command line based approaches [21], and far more accurate than call-sets from any individual tool. Importantly our system exhibits better sensitivity and higher specificity than equivalent commercial software.

CoVaCS is available through a user-friendly web interface at: https://bioinformatics.cineca.it/covacs and is currently hosted at Cineca (http://www.hpc.cineca.it/content/about-us) on the Pico infrastructure - an Intel Cluster of 74 nodes built for large-scale analysis - and benefits from all the advantages of High Performance computing (HPC).

## Implementation

CoVaCS is currently hosted at PICO: a High Performance Data Analytics Linux Cluster with 80 Intel NeXtScale nodes, integrated within a multi tier storage system, configured with 50 TBytes of SSD memory, 5 PBytes of High IOPS storage and tens of PBytes of long term archiving library.

### Web Interface

The web interface is based on the Foundation front-end framework v. 5.0 and Javascript/jQuery. Server-side, the system is served by the Apache HTTP Server v. 2.4.6 on CentOS and is based on several in-house PHP v. 5.4 scripts for the management of complex data and results. Data are stored in a MySQL DBMS v. 5.5. Variant detection, consensus call-set assessment and functional annotation is carried out by state of the art tools as detailed in Additional file 1.

CoVaCS accommodates all major commercial exome sequencing kits (Illumina, Agilent or Nimblegen) and includes a collection of the most recent assemblies for human, mouse and cow genomes with corresponding annotations.

### Tools incorporated in CoVaCS

Trimmomatic [22] (version 0.33) is used for initial quality trimming of the reads. Quality reports of pre- and post-trimming data are generated by the means of the Fastqc [23] program. Reads can be aligned to reference genomes by Bowtie2 [24] or bwa [25]- Sorting and compression of alignment files is performed with SAMtools [26] utilities.

PCR duplicate removal is performed using the MarkDuplicates utility from the Picard [27] tool suite (version 1.119).

Varscan [17], the GATK [18] best practice pipeline, [19] employing the HaplotypeCaller and Freebayes [20] are used for variant calling.

Integration of call-sets is performed by the means of a custom script in combination with the CGES [21] consensus genotyper.

Functional annotation for the prediction of causative mutations is performed using Annovar [28] and a collection of databases and publicly available resources of human genetic variation, including dbSNP [29], Omim [30], Cosmic [31] and Clinvar [32] among others.

A detailed description of the implementation of the pipeline and incorporated custom utilities is reported in Additional file 1.

Vcf files for CGES were generated using Freebayes [20], GATK [19], Atlas SNP2 [33] and SAMtools [26] with the same parameters as specified in the supplementary materials of Trubetskoy et al.

## Results

**Fig. 1:**
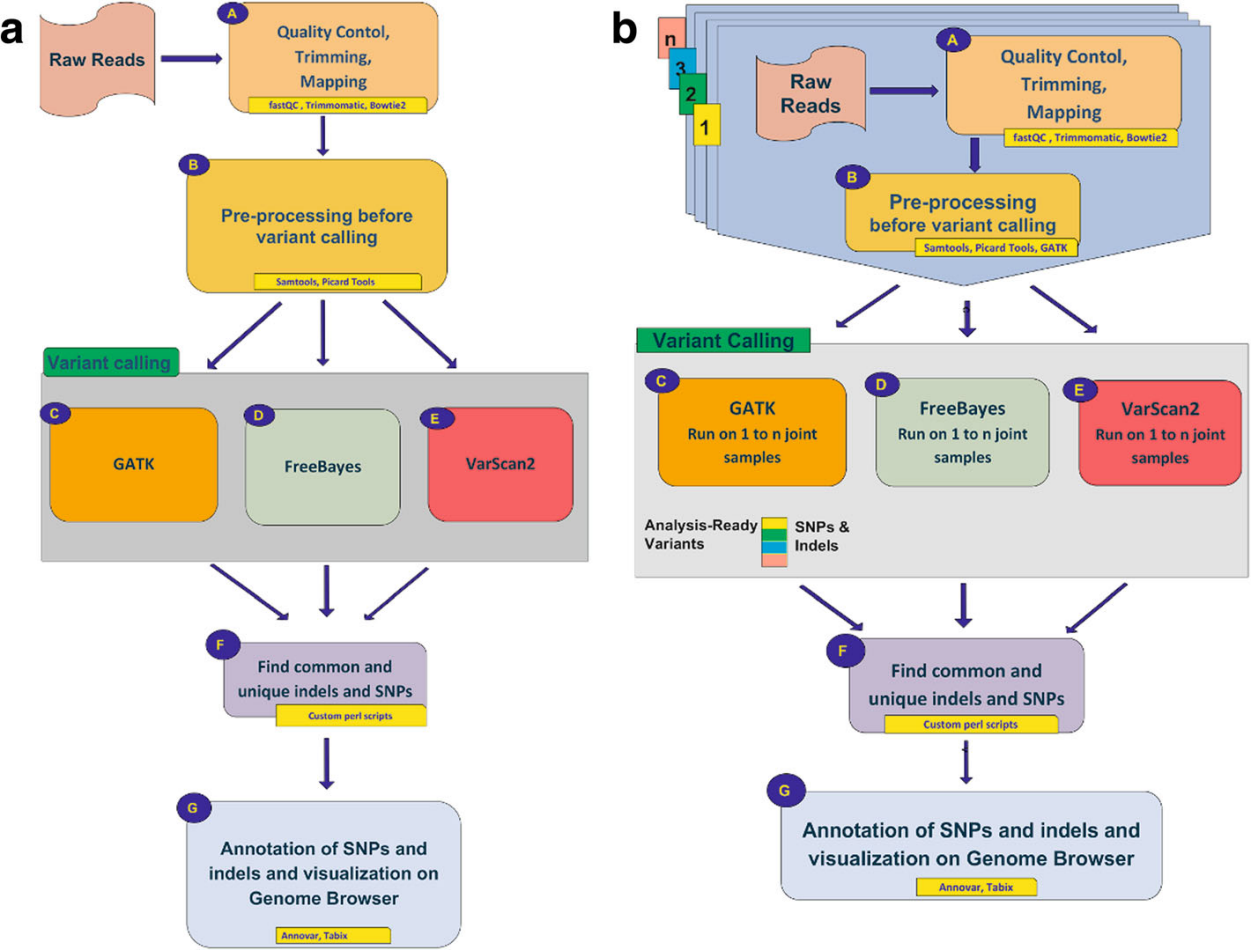
Schematic of the variant calling pipelines implemented in CoVaCS: Single steps of the pipelines are indicated by capital letters: A to G. Tools are indicated in yellow boxes. **a** Single sample variant calling, **b** Joint sample variant calling

CoVaCS consists of a powerful and user friendly web interface providing access to computational resources and state of the art tools and pipelines for the analysis of genome re-sequencing data. The system, currently incorporates different assembly versions and a collection of annotations for the human (hg18, hg19, hg38), mouse (mm9, mm10), and cow (bostau7, bostau8) genomes, as well as a collection of meta-data for the major commercial exome kits by Illumina, Agilent and Nimblegen. Custom targeted resequencing panels can be analyzed by simply uploading a file in bed format, specifying the coordinates of targeted regions. Pipelines for joint- (*jsp*) and single-sample (*ssp*) variant calling are provided, allowing the analysis of both large or limited cohorts of samples. Both pipelines are composed of 8 steps and accept either fastq files or alignment files in bam format as their main input. When a bam file is provided, the alignment indicated in the bam file is used in the subsequent stages of the analysis. A schematic is presented in Figs. 1a for *ssp.* and 1B for *jsp.* An extended description of the computational steps and parameters is provided in Additional file 1.

Accurate genotyping is achieved through the combination of three variant calling algorithms: GATK haplotype caller [18, 19] Varscan2 [17] and Freebayes [20].

The final call-set is a simple majority rule (2 out of 3 tools) consensus from the individual call-sets, while low confidence calls (specific to one tool) are reported as a separate call-set.

The main output consists of a detailed HTML report, containing the predicted variants, along with their functional annotation against a large collection of publicly available resources and databases, including dbSNP [29], Omim [30], Cosmic [31] and Clinvar [32]. Dynamic filters can be used to sort results and prioritize candidate causal variants. Various output formats (vcf, xls, csv, txt) can be generated and all intermediate files - including call-sets obtained by each variant calling algorithm - are available for download. UCSC genome browser track files are included in the final output to facilitate visualization of results.

### Data input, control management and variant filtration

Submission to CoVaCS requires a structured description of the experimental design of a project through the creation of a “study”. Studies are high level containers for sequencing data and meta-data- including sample labels, experimental conditions, biological replicates. There is no limitation on the number of samples and files that can be incorporated into a study. and users are guided in the process of study creation and population through a series of HTML forms.

CoVaCS accepts the most common file formats for sequencing (fastq) and alignment (bam/sam) data. Compressed files and archives (sra, zip, gz, tar) are also supported. Files can be uploaded directly through the web interface (if smaller than 2Gb), or by the means of ftp, dropbox or weblink. Prior to the submission of an analysis, a reference genome and exome capture kits or a manifest file containing chromosome coordinates in bed format (where appropriate) can be selected from a drop-down menu.

The execution of the workflows is controlled through a dedicated “monitor” application which allows the retrieval of intermediate output files and status checks on submitted jobs. Upon completion, an email provides the user with links to the final output, which consists of a dynamic html report with the complete list of variants and their functional annotations, including the class of the variants (SNV/INDEL), gene names and genomic coordinates, genotypes and, where available, accession numbers from a collection of repositories of genetic variation, such as dbSNP, ExAC and 1000 genomes [6, 7, 29]. Comprehensive report files also contain detailed information concerning the execution of each step, read qualities and mapping, as well as statistics regarding predicted variants.

Several filters can be applied to the final results in order to facilitate variant prioritization and the identification of potentially causative mutations. Filtering criteria can be specified through a user-friendly interface by selecting the appropriate filters from a drop-down menu.

For example, the “Functional class” column allows users to filter variants according to their functional class as defined by Annovar. The “NM” column, which reports the number of methods supporting each variant can be used to filter variants called by particular combinations of variant calling methods. Variants can also be filtered according to their MAF (minor allele frequency), as estimated from 1000G project [6], or based on their class (SNVs or indels), or their presence or absence in databases of human genetic variation, such as dbSNP. Moreover, users may select variants falling within particular genes or genomic regions, which can facilitate the analysis of disease causing mutations in monogenic disorders. Results, filtered according to the specified criteria, are available for download in different formats (simple text and excel xlsx).

### Evaluation of CoVaCS variant calling pipeline

In order to evaluate the accuracy and the sensitivity of the pipelines incorporated into CoVaCS, we have taken advantage of a set of publicly available exome (WES) and genome (WGS) resequencing data, derived from the Platinum genome NA12878 [34], to compare results attained by our tool with the proprietary Illumina VCAT2 software, and CGES (Consensus Genotyper for Exome Sequencing) [21], a similar variant calling workflow based on a consensus strategy. Accuracy estimates were derived for each tool by comparing the complete set of predictions with genetic variants as indicated in the reference vcf for the NA12878 platinum-genome. Consistency of the calls was evaluated at genotype level. Only calls showing complete agreement with the validation set were considered correct.

The core of the CGES package consists of a collection of command line utilities for the harmonization of VCF files, obtained by the means of different variant calling algorithms. Consensus genotypes are obtained by applying a two-stage voting scheme based on user defined cut-offs values. In the first step only variants that are detected by a minimum number of tools are selected. Subsequently a genotype concordance threshold is applied and inconsistent genotype calls that are not supported by a minimum number of algorithms are marked as discordant. The final call-set consists of a collection of high quality variants that show consistent genotype calls according to the required number of algorithms.

Although in principle the tool could be applied to any combination of vcf files, authors of CGES have developed their own variant calling pipeline based on the integration of 4 popular variant calling algorithms: GATKv2.8, SAMtools, Altas-SNP2 and FreeBayes. According to the authors, best results in terms of specificity for CGES are achieved when strict consensus of all calls (4 methods) is applied. Parameters for the optimization of the suggested variant calling algorithms with CGES are also provided.

WES data analyzed in the course of the current evaluation derive from a pilot study performed by Illumina in order to demonstrate the accuracy and sensitivity of commercial VCAT2 variant calling system. The data were produced with the Nextera Rapid Capture exome kit (https://blog.basespace.illumina.com/2015/01/14/variant-calling-assessment-using-platinum-genomes-nist-genome-in-a-bottle-and-vcat-2-0/), and provide sets of different levels of exome coverage: 50X, 100X, 220X and 400X to allow tests of coverage on the accuracy and sensitivity of genotyping. All the original sequencing data, as well as the final vcf are publicly available on the Illumina basespace portal (https://basespace.illumina.com/s/JU28qsbkN1vS).

In the present work the 50X, 100X and 220X pooled coverage sets were analysed with CoVaCS, using the default parameters and the bwa aligner. The CGES pipeline was executed, using its default parameters, on the same alignment files generated by bwa in order to facilitate direct comparison of the results. Both a strict consensus based on concordance of all the 4 methods and a less stringent consensus requiring the support of at least 2 out of the 4 methods were produced.

**Fig. 2:**
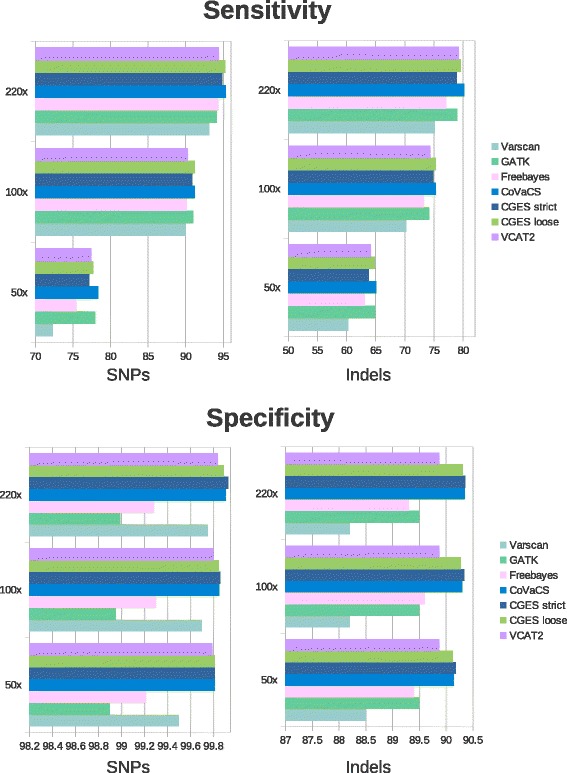
Comparison of variant calling algorithms on WES data. Sensitivity and specificity, at varying levels of coverage, of variant detection algorithms used in the course of the present study, in the analysis of the golden standard WES benchmark based on the NA12878 platinum genome

Results in terms of sensitivity and specificity (wrt the platinum genome call-set obtained from https://cloud.google.com/genomics/data/platinum-genomes) are reported in Fig. 2 and Additional file 2: Table S1 and suggest that, as expected, methods combining the predictions of different variant calling algorithms, such as CoVaCS and CGES achieve a substantially higher sensitivity and specificity than any of Varscan, GATK or Freebayes taken individually, confirming that CoVaCS provides a highly accurate pipeline for the genotyping of WES data. These considerations apply also for the Illumina VCAT2 software which attains a lower sensitivity, with respect to the consensus methods, both for SNVs and indels. Unsurprisingly, in the light of the similar approaches (and selection of tools) underlying the two workflows, results attained by CoVaCS and CGES on this data-set are substantially equivalent, and differ only by a limited number of calls. However, we notice that for CGES, the call-set based on the strict consensus achieves a somewhat lower sensitivity than the equivalent set based on a 2 out 4 rule, without showing any particular improvement of the specificity. In our hands, CoVaCS displays a marginal, but systematic increase in sensitivity with respect to both the CGES call-sets. This observation is particularly evident when the “low coverage” 50X set is considered.

High coverage PCR free whole genome sequencing data for the NA12878 platinum genome were downloaded from http://www.ebi.ac.uk/ena/data/view/PRJEB3381 (50×) and http://www.ebi.ac.uk/ena/data/view/PRJEB3246 (200×).Data were subjected to the CoVaCS single-sample variant calling pipeline. As for WES data, the CGES pipeline was executed on the same alignment files and 2 equivalent consensus call-sets (4 out of 4 and 2 out of 4 rules) were obtained also for the WGS analysis. Results in terms of specificity and sensitivity were evaluated considering only genomic regions encompassed by at least 10 uniquely mapped reads (88% of the genome for 50× and 96% for 200×).

**Fig. 3:**
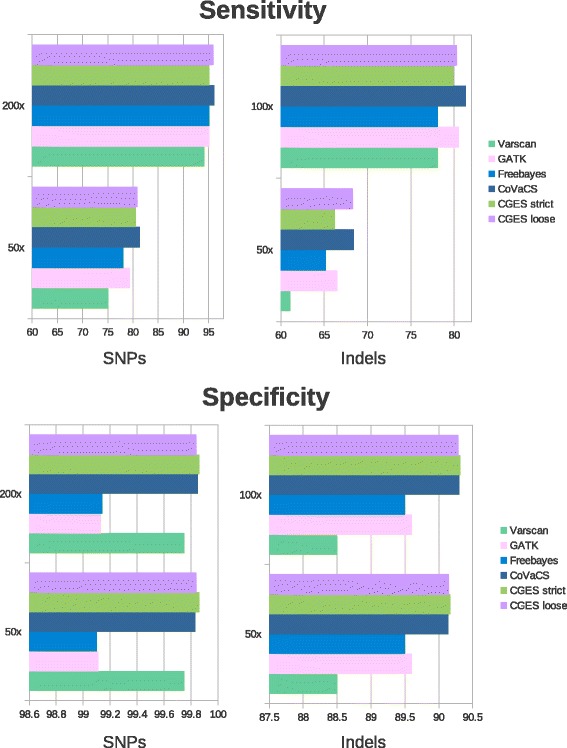
Comparison of variant calling algorithms on WGS data. Sensitivity and specificity, at high (200X) and low (50X) levels of coverage, of variant detection algorithms used in the present study, for the analysis of the golden standard WGS benchmark based on the NA12878 platinum genome

Consistent with previous observations, CGES and CoVaCS attain a higher sensitivity and specificity than call-sets based on single tools (Fig. 3 and Additional file 2: Table S2). As expected, the 2 also tools attained equivalent results also on this data-set, with a minimal difference in the total number of correct calls. We notice, however, that also in this case CoVaCS displays the same specificity and a marginal increase in sensitivity with respect to both CGES call-sets. Consistent with previous observations, the difference is more marked when the strict consensus set is considered. Importantly, and again in line with previous observations, the increase in sensitivity is more evident for the low coverage set, suggesting a recurrent pattern.

**Fig. 4:**
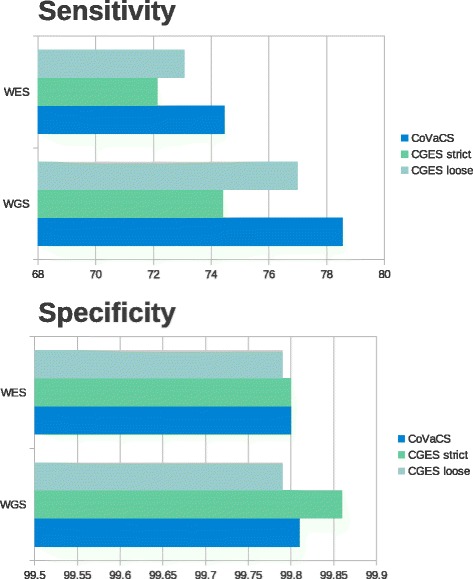
Comparison of variant calling CGES and CoVaCS on regions of low coverage. Comparison of accuracy and specificity levels achieved by the CoVaCS and CGES on genomic regions encompassed by less than 30 uniquely mapping reads, for the WES (A) and WGS (B) data

In order to investigate this scenario, we decided to compare predictions by CGES and CoVaCS on shallow coverage regions, that were encompassed by less than 30 uniquely mapped reads. Direct comparison of the predictions with the golden standard benchmark (Fig. 4 and Additional file 2: Table S3), again highlight a more relevant difference in specificity between CGES and CoVaCS when only low coverage regions are considered. This observation applies both for WES and WGS data.

Since the 2 workflows are based on a similar selection of tools, which are however executed using different parameters, we speculate that this difference is probably related to the usage of more stringent parameters for variant calling in the CGES pipeline.

Interestingly, we observe that the call-set for exome target regions obtained from whole genome data is more accurate than the equivalent set derived from exome data, even at lower nominal coverage levels, consistent with the findings of [35]. The higher accuracy is probably related to a more uniform coverage profile (Additional file 2: Table S4 and Additional file 3: Figure S1), as we observe a considerable reduction in coverage of GC rich regions in the WES data.

Evaluation of the pipeline on non human data (see Additional file 1) confirm our observations and suggest that the pipeline implemented in CoVaCS can be also be applied to the analysis of non human model systems (Additional file 2: Table S5).

### Comparison of the variant calling algorithms incorporated in CoVaCS

Interesting patterns, often corresponding to previous suggestions [36], emerge from the analysis of false positive and false negative calls recovered (or missed) by the variant calling algorithms incorporated in CoVaCS. For GATK, we observe a tendency to over predict variants that were incorporated in the original training set (8% of the false positive calls fall in this class), however since this tool adopts a set of variant filtering criteria that are not solely driven by coverage, GATK recovers a better sensitivity than Freebayes or Varscan in low coverage regions (Additional file 2: Table S1), especially in the detection of heterozygous variants. Freebayes shows the best overall performance among all the tools used in our experimental setup. However, we observe a reduction in sensitivity for this method in low coverage regions and especially in the detection of heterozygous variants. This is particularly apparent for the WES data-set (Additional file 2: Table S2) due to both the lower uniformity in coverage and to the so called “reference capture bias”, where capture probes tend to preferentially enrich reference alleles at heterozygous loci. Among the tools used in CoVaCS, Varscan shows the highest specificity at the cost of a slight decrease in sensitivity. As for Freebayes low coverage is the main factor affecting the specificity of Varscan. Importantly, we notice that the false positive rate remains relatively low for all the call-sets produced in the course of this study. The majority of these false positives are likely to derive from erroneous mapping of the reads or errors in the reference genome assembly rather than flaws in the variant calling algorithms. Indeed, consistent with Zook et al., 2014 [37], we observe that a notable proportion of the reads (21%) from which such erroneous calls originate correspond to genomic regions of low mappability (average mappability below 0.25 according to the GEM tool [38]) or map to different genomic locations on hg19 and hg38 reference genomes (37%).

## Conclusions

Comparative analyses based on publicly available WES and WGS sequencing data for the golden standard platinum genome NA12878, show that the CoVaCS consensus calls for WES targeted resequencing data are slightly more sensitive and notably more specific than those generated with the Illumina VCAT 2.0 software or those generated by the means of any individual predictors. Detailed comparisons with a similar workflow based on a consensus strategy, CGES, show that performances attained by CoVaCS are completely in line with those attained by equivalent methods, with a marginal but systematic increase in sensitivity in regions of shallow coverage.

CoVaCS is accessible through a dedicated, user-friendly web interface and no configuration or installation is required. All steps, from quality trimming to variant annotation can be performed, for both single- and joint-samples by non-specialists.

The system is currently hosted at Cineca and offers the speed of a HPC computing facility, a crucial consideration when large numbers of samples must be analysed.

CoVaCS is freely available to all Cineca Users, while Members of European research institutions can obtain full access to CoVaCS by applying through e ELIXIR-IIB (Italian Infrastructure for Bioinformatics, http://elixir-italy.org/). The system is under constant development and new reference genomes, databases and bioinformatics tools are frequently added to it. Importantly, all the analyses are performed automatically allowing high reproducibility of the results. As such, we believe that CoVaCS can be a valuable tool for the analysis of human genome resequencing studies.

## Additional files

Additional file 1: Supplementary methods and results. (DOC 57 kb)

Additional file 2: Supplementary Tables S1 to S5 (XLS 18 kb)

Additional file 3: Supplementary Figure S1 (TIFF 127 kb)

## Abbreviations

JSP: Joint sample
SNV: Single nucleotide variant
SSP: Single sample
TGS: Target gene sequencing
WES: Whole exome sequencing
WGS: Whole genome sequencing
