# Supplementary material for: CoVaCS: a consensus variant calling system
Source: BMC Genomics. 2018 Feb 5;19:120. doi: 10.1186/s12864-018-4508-1 (PMC5800023; doi:10.1186/s12864-018-4508-1)
Supplement: Supplementary file 1 — Supplementary methods and results. (DOC 57 kb) [file 12864_2018_4508_MOESM1_ESM.doc]

**Detailed description of CoVaCS pipelines**

**Quality preprocessing and outline**

After preprocessing of data using Trimmomatic [1] program (version 0.33) reads are aligned to genomic indexes retrieved from the Illumina i-genomes repository (<http://support.illumina.com/sequencing/sequencing_software/igenome.html)> with Bowtie2 [2]. PCR duplicates are removed using the MarkDuplicates utility from the Picard [3] tool suite (version 1.119)and sorting and compression of alignment files is performed with Samtools [4] utilities.

CoVaCS provides pipelines for both joint- (*jsp*) and single-sample (*ssp*) variant calling. The *jsp* procedure allows analysis of a batch of samples, integrating population or pedigree information in the prediction of variants. Joint analysis typically exhibits improved sensitivity for low frequency variants as well as improved specificity at loci with low sequence coverage. Genotype calls are emitted for all loci where at least one individual in the cohort is polymorphic [5, ], facilitating direct comparison of genotypes. The ssp workflow which employs only data from single individuals, is appropriate for individual and small-scale studies. When resequencing coverage is relatively uniform and of adequate coverage, ssp procedures show similar sensitivity and specificity to joint-sample calling strategies, although performance can be impaired when these conditions are not satisfied [6].

**STEP A:** Quality trimming

Since variant calling algorithms are more robust to sequencing errors than to reduced coverage, relatively relaxed sequential pre-processing steps are performed using Trimmomatic [1]).

- Any adapter sequences in the sequence data are removed
- The “Leading” flag removes nucleotides from the 5' end of the reads if their quality score falls below a predefined cutoff (Q<=5)
- “Trailing” which performs the equivalent operation on the 3' end of reads
- “Slidingwindows” evaluates the average quality scores of the reads along sliding windows of fixed length (4 bp), cutting the read once the average quality within the window falls below a threshold (Q<=15)
- Reads shorter than 50 bps after trimming discarded and the fastQC program used to generate diagnostic reports on trimmed and untrimmed reads to facilitate evaluation of the efficacy of pre-processing steps.

**STEP B**: Alignment and Alignment post processing

Reads are aligned to the reference genome using Bowtie2 [Langmead et al, 2009] with the “sensitive” pre-sets.

The sam format provided by Bowtie2 is converted to the binary Alignment/Map format (BAM) and sorted by chromosomal coordinates using SAMtools [4]. The Picard [3] markDuplicates utility is used to remove possible PCR duplicates which are a substantial source of sequencing bias and can introduce systematic errors in the variant calling process. BAM files are then indexed using SAMtools.

For the GATK pipeline alone, BAM files are subjected to base quality score recalibration (BSQR) prior to variant calling, following GATK best practices. BSQR is an empirical procedure based on machine learning techniques that model sequencing errors and adjust quality scores . The GATK BaseRecalibrator program generates an error model from the estimated empirical error frequencies in the alignments and adjusts the quality scores to represent true error probabilities more accurately. Polymorphic sites included in publicly available databases and collections of genomic variants are excluded to avoid over-correction.

CoVaCS submits processed BAM files to 3 different variant calling algorithms (STEPS C,D, and E)

**STEP C GATK Haplotype caller.** The GATK HaplotypeCaller [7]

adopts a variant discovery algorithm based on local reassembly of the reads. Polymorphic genomic regions are identified from the alignment files, and reads mapping to such regions are subjected to targeted reassembly in order to reconstruct the “local” haplotypes. Genetic variants are identified from the reconstructed sequences (haplotypes) and SNPs and indels are detected concurrently, directly from the assembly. This strategy can greatly improve the accuracy of variant detection in highly polymorphic regions, where, due to high levels of variability reliable alignment of the reads to the reference genome is often problematic. In the joint-sample procedure individual gVCF files obtained from the HaplotypeCaller program, are combined into a multi-sample gVCF using the GATK GenotypeGVCF program.

Variant filtering for the identification of high-confidence variants is performed, both for the joint and the single sample analyses, according to the GATK best practice Variant Quality Score Recalibration procedure. The Variant Recalibrator estimates an adaptive model based on a set of high quality "true variants" (recovered from publicly available resources) in order to identify an optimal combination of quality filtering metrics and the final vcf file containing the final set of high-confidence variants is produced by the means of the GATK ApplyRecalibration program, using 99 and 98.5 tranches for SNPs and indels respectively.

**STEP D Freebayes**

Freebayes [] adopts an adaptive probabilistic model, based on Bayes theorem, for variant discovery. Local haplotype reconstruction (but not reassembly) based on read extension and permutation increases the sensitivity of the approach and facilitates the detection of false positive variants deriving from “unlikely” haplotypes incorporating mismatches that are likely to represent inaccurate mapping of the reads or sequencing errors. Different variables (number of individuals, ploidy of the genome, copy number of genomic regions) can be incorporated into the model, in order to generate more accurate prior probabilities. Variant calling is performed using a “simple” probabilistic model to reconstruct the most-likely combination of genotypes in the sample. Since no machine learning procedure-based on golden standard variant datasets-is required, Freebayes achieves similar levels of accuracy for genomes where large collections of known variants are not available. In CoVaCS, VCF files obtained by Freebayes are subjected to a simple hard-filtering (as suggested by the developers of the program) and all variants with a quality lower than 20 are removed from subsequent steps. Any number of bam files can be provided as input. and no particular configuration is required for the joint or single-sample variant calling.

**STEP E**: VarScan2 method (2.3.7):

VarScan2 [9] applies a set of robust quality filters and heuristics for the detection of high confidence variants. Ambiguously mapped reads are discarded and thresholds for coverage, quality, variant frequency, and/or number of reads required to call a variant are applied in order to identify a set of reliable calls. Strategies based on hard filters are less likely to be confounded by factors such as extreme read coverage or sample contamination and therefore are more robust than strategies based on machine learning or Bayesian statistics under such circumstances [9]. Varscan2 requires a pileup file as input (generated by SAMtools) and can output various formats, including vcf.

The program can analyse any number of input files concurrently and no special configuration is required for the joint or single sample variant calling procedures. A simple hard-filtering step accepts only variants with quality scores (QD) over 20 as high-confidence predictions.

**STEP F**: Consensus call-set. Predictions from Varscan2, GATK and Freebayes are consolidated into a single call-set following a simple majority consensus rule. Variants identified by at least two methods are incorporated into a final “high confidence” call set. Singleton variants predicted by only one method are considered less reliable and are included in a low quality call-set, which is also available for download. VCF file intersection is performed by a custom Perl script. The final output consists of four VCF files:

- SNPs common to at least 2 methods
- INDELs common to at least 2 methods
- Singleton SNPs recovered by only one method
- Singleton INDELs obtained from a single method

**STEP G**: Annotation of SNPs and indels and visualization on the Genome Browser

Functional annotation of variants is performed by Annovar (v. 2016 Feb 01) [10] Human variants are compared to an extensive ensemble of databases and publicly available resources including 1000 Genomes [11] and Exac03 [12] to provide information on the prevalence of the variants in different populations, CADD [1312], DANN [14] and Spidex [15] to evaluate the functional impact of non-synonymous and splice site mutations and specialized databases of disease causing mutations including Clinvar [16]. For species where such a wealth of information is not available, functional annotation of the variants is performed against the Refseq annotation and variant frequencies are recovered from the appropriate dbSNP version [17]. The final vcf files are indexed using the tabix utility [18] (v. 0.2.6) and UCSC genome browser tracks are provided to facilitate their visualization. A custom script (WesParser) is used to incorporate calls from all the VCF files and relevant annotations into a interactive HTML report, that constitutes the main output of CoVaCS. Dynamic filters can be used to sort results and prioritize candidate causal variants and filtered variants are available for download in various output formats (xls, csv, txt).

<https://www.ncbi.nlm.nih.gov/pubmed/27622383> topo

**User Registration**

Users are required to register at [https://bioinformatics.cineca.it/covacs/](https://bioinformatics.cineca.it/ivacs/). Registration is free of charge for academic/research purposes. Members of European research institutions can obtain full access to CoVaCS by applying through ELIXIR-IIB (Italian Infrastructure for Bioinformatics, http://elixir-italy.org/). Please write to [hpc-bioinformatics@cineca.it](mailto:hpc-bioinformatics@cineca.it) for further information. The system adopts a strict access management policy in order to enforce data security and protection. Access to the data is exclusive and private for individual users and data can be shared only between CoVaCS users registered within the same project.

**Submission forms**

Submission to CoVaCS requires a structured description of the experimental design and the definition of a series of parameters through a series of HTML forms consisting of the following steps.

**1. Creation of a study** - A study is a high (top) level container storing all the information related to a sequencing project. Studies in CoVaCS can contain any number of sequencing experiments and samples, accompanied with their corresponding metadata (labels, experimental condition, biological replicates). Samples belonging to the same study are usually part of the same research project. There is no restriction on the number of analyses that can be applied to samples belonging in the same studies, results of such analyses will always be associated with the corresponding study.

**2. Sample upload and metadata definition** - Following the creation of a study, sequencing files are uploaded to CoVaCS. CoVaCS offers four different means for uploading data files into the system. Files of less than 2 GB in size can be uploaded directly through the web interface, while files surpassing this size limit can be uploaded by the means of ftp, dropbox or weblink. Samples can be associated to any number of studies. CoVaCS supports the following file formats:Sequencing files (fastq)

- Alignment files (bam)
- Variant calling format files (vcf)
- Compressed archives (sra, zip, gz, tar).

Paired-end sequencing data need to be correctly matched during the upload. Sample names, which are required to be unique, must be provided for all the samples and any type of metadata describing the sample and the sequencing process can be associated with the files

**3. Analysis selection** – This stage requires the selection of the variant calling procedure to be used: single sample or joint. Users are required to specify the appropriate reference genome, to indicate the kit used for library construction (for the removal of the adapter sequences) and -in the case of WES- to select the proper enrichment kit. Targeted resequencing studies with custom designs require the upload of a bed file specifying the coordinates of the target regions. For targeted resequencing studies user can specify the amount of padding of the target regions (default 100bp).

Upon completion an email provides the user with links to the final output that consists of a dynamic html report with the complete list of variants and their functional annotations, including the class of the variants (SNV/INDEL), gene names and genomic coordinates, genotypes and, where available, accession numbers from a collection of repositories of genetic variation, such as dbSNP.

**Evaluation of CoVaCS on a non human model system.**

In order to evaluate the performances of CoVaCS on a set of non human genome resequencing data, we have taken advantage of a recent study reporting genotyping data for 13 inbred laboratory mouse strains [20]. Raw reads data, as available from the European Nucleotide Archive (Project id ERP000927), were downloaded and mapped to the reference mm10 genome, by the means of the bwa-mem program. A total of more than 18 billions reads were mapped to the mm10 reference. The average coverage ranged in between 73 (C57BL/10J) to 147X (ST/bJ). Variant calling was performed as for the human data, by running the CGES and CoVaCS pipelines with default parameters, on the same alignment files. Analogously to the analysis of human data, two CGES consenus call-set were produced, one, more stringent, requiring the complete consensus of the 4 variant callers, and the second one, more relaxed, based on the minimum agreement of at least 2 tools.

Evaluation of the accuracy and sensitivity of variant calling pipelines, was carried out, as in the original paper, by comparison with a set of 138000 high quality genotypes reported by the HapMap consortium [19]. Consistent with what already observed for the human platinum-genome, methods based on consensus strategies, such as CGES and CoVaCS achieved substantially better performances than any of the individual callers considered in this study. Consistent with what already observed in the analysis of the human data, the CGES pipeline based on full consensus shows a slight decrease in sensitivity with respect to both CoVaCS and the alternative CGES pipeline based on more lrelaxed consensus rule. Importantly this comes with no detectable increase in specificity, suggesting that in our hands, the CGES pipeline based on full consensus is probably too stringent. The CGES pipeline based on a loose consensus rule and CoVaCS show a very similar performance on this dataset with no detectable difference in the estimated levels of accuracy. All in all these results are highly consistent with our previous observations.

**Supplementary References**

[1] Bolger AM, Lohse M, Usadel B. (2014). Trimmomatic: a flexible trimmer for Illumina sequence data. *Bioinformatics*. 30(15):2114-2120.

[2] Langmead, B, Trapnell, C, Pop, M, Salzberg, SL. (2009). Ultrafast and memory-efficient alignment of short DNA sequences to the human genome. *Genome Biology*, 10 (3), pp. R25

[3] Wysoker, A, Tibbetts, K, Fennell, T. (2013). Picard tools version 1.90 http://picard.sourceforge.net

[4] Li H, Handsaker B, Wysoker A, et al. (2009). The Sequence Alignment/Map format and SAMtools. *Bioinformatics*.;25(16):2078-2079.

[5] Van der Auwera GA, Carneiro M, Hartl C, et al. (2013), From FastQ Data to High-Confidence Variant Calls: The Genome Analysis Toolkit Best Practices Pipeline. *Current Protocols in Bioinformatics.* 11(1110), 11.10.1–11.10.33.

[6] Nho K, West JD, Li H, et al. (2014). Comparison of Multi-Sample Variant Calling Methods for Whole Genome Sequencing. *IEEE International Conference on Systems Biology : [proceedings] IEEE International Conference on Systems Biology*. 2014:59-62.

[7] McKenna A, Hanna M, Banks E, et al. (2010). The Genome Analysis Toolkit: a MapReduce framework for analyzing next-generation DNA sequencing data. *Genome Research*, 20(9):1297–1303.

[8] Garrison E, Marth G. (2012). Haplotype-based variant detection from short-read sequencing. ArXiv:1207.3907

[9] Koboldt DC, Zhang Q, Larson DE, et al. (2012). VarScan 2: Somatic mutation and copy number alteration discovery in cancer by exome sequencing. *Genome Research*, 22(3): 568–576

[10] Li H. (2011). A statistical framework for SNP calling, mutation discovery, association mapping and population genetical parameter estimation from sequencing data. *Bioinformatics.* (21):2987-93

[11] The 1000 Genomes Project Consortium. (2015), A global reference for human genetic variation. *Nature*. 526(7571):68-74

[12] Exome Aggregation Consortium. (2016). Analysis of protein-coding genetic variation in 60,706 humans. *Nature*. 18;536(7616):285-91.

[13] Kircher M, Witten DM, Jain P, O'Roak BJ, Cooper GM, Shendure J. (2014). A general framework for estimating the relative pathogenicity of human genetic variants. *Nature Genetics*, 46:310–315

[14] Quang D, Chen Y, Xie X. (2015). DANN: a deep learning approach for annotating the pathogenicity of genetic variants. *Bioinformatics*, 31(5):761–763.

[15] Xiong, Hui Y et al. (2015). The Human Splicing Code Reveals New Insights into the Genetic Determinants of Disease. *Science* . 347:6218,

[16] Landrum MJ, Lee JM, Riley GR, et al. (2014). ClinVar: public archive of relationships among sequence variation and human phenotype. *Nucleic Acids Research*. 1;42(1).

[17] Sherry ST, Ward MH, Kholodov M, et al. (2001). dbSNP: the NCBI database of genetic variation. *Nucleic Acids Research*, 29(1): 308–311.

[18] Li H. (2011). Tabix: fast retrieval of sequence features from generic TAB-delimited files. *Bioinformatics* 27 (5): 718-719.

[19] Kirby A, Kang HM, Wade CM, Cotsapas et al. (2010). Fine mapping in 94 inbred mouse strains using a high-density haplotype resource. *Genetics.* 185:1081–95.

[20] Doran AG, Wong K, Flint J, Adams DJ, Hunter KW, Keane TM. (2016). Deep genome sequencing and variation analysis of 13 inbred mouse strains defines candidate phenotypic alleles, private variation and homozygous truncating mutations. *Genome Biology*. 17:167.

.

.
