# Supplementary figures and images for: CoVaCS: a consensus variant calling system
Source: BMC Genomics. 2018 Feb 5;19:120. doi: 10.1186/s12864-018-4508-1 (PMC5800023; doi:10.1186/s12864-018-4508-1)

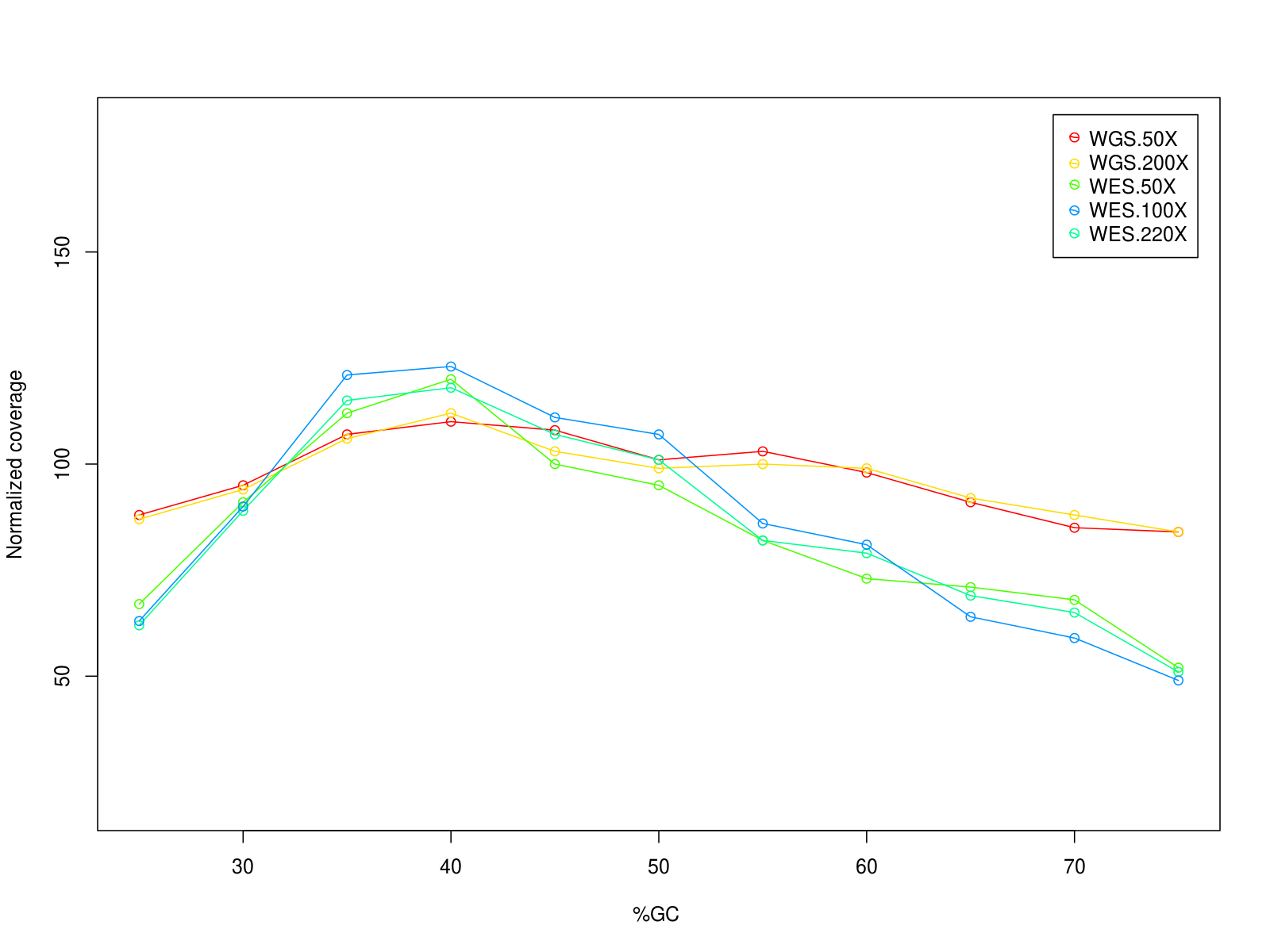

Supplement: Supplementary file 3 — Supplementary Figure S1 (TIFF 127 kb) [file 12864_2018_4508_MOESM3_ESM.tiff]
